# Role of Artificial Intelligence in Kidney Disease

**DOI:** 10.7150/ijms.42078

**Published:** 2020-04-06

**Authors:** Qiongjing Yuan, Haixia Zhang, Tianci Deng, Shumei Tang, Xiangning Yuan, Wenbin Tang, Yanyun Xie, Huipeng Ge, Xiufen Wang, Qiaoling Zhou, Xiangcheng Xiao

**Affiliations:** 1 Department of Nephrology, Xiangya Hospital, Central South University, 87 Xiangya Road, Changsha, Hunan 410008, China; 2 Department of Nephrology, Second Affiliated Hospital of Soochow University, 1055 Sanxiang Road, Suzhou, Jiangsu 215000, China

**Keywords:** Artificial intelligence, kidney disease, Alerting systems, Diagnostic assistance, Guiding treatment, Evaluating prognosis

## Abstract

Artificial intelligence (AI), as an advanced science technology, has been widely used in medical fields to promote medical development, mainly applied to early detections, disease diagnoses, and management. Owing to the huge number of patients, kidney disease remains a global health problem. Challenges remain in its diagnosis and treatment. AI could take individual conditions into account, produce suitable decisions and promise to make great strides in kidney disease management. Here, we review the current studies of AI applications in kidney disease in alerting systems, diagnostic assistance, guiding treatment and evaluating prognosis. Although the number of studies related to AI applications in kidney disease is small, the potential of AI in the management of kidney disease is well recognized by clinicians; AI will greatly enhance clinicians' capacity in their clinical practice in the future.

## Introduction

Kidney disease is a major public health problem in part because of its common etiology caused by diabetes, hypertension, obesity, and aging; the incidence of these conditions is increasing. According to the Global Burden of Diseases, Injuries, and Risk Factors Study 2015, 750 million people worldwide suffered from kidney disease 1. Kidney disease brings a huge burden to society. In 2017, a survey showed that the yearly cost was approximately $1,205 for a patient with stage 3 chronic kidney disease (CKD3), $1963 for a CKD4 individual, $8,035 for a person at CKD5 condition, and $34,554 for a hemodialysis patient 2. Early detection and prevention of the progression of kidney disease towards end stage renal disease is therefore of significant importance.

AI is a science of computer simulated thinking processes and human behaviors, which involves computer science, psychology, philosophy and linguistics. In 2016, Alphago 3-0 won a professional human Go player. It was the first computer program that defeated a world champion of Go, clearly revealing the potential that AI will bring technological advances in the era. The continued rapid growth in computer-processing power over the past two decades, the availability of large data sets and the development of advanced algorithms have driven major improvements in machine learning^3^.

Electronic medical records (EMR) provide large-scale and real-world clinical data, which is the basis for developing AI technology in the clinic. It is challenging for humans to directly analyze these massive data; this is not only because of the massive time required and cares needed to avoid human errors but also the ability to derive the insights or information in depth. Clearly, AI technology holds nonparallel advantages over humans in these domains 4. The studies of AI in kidney diseases are at a beginning stage. According to the existing literature, the function of AI in kidney disease mainly focuses on four aspects: Alerting systems, Diagnostic assistance, Guiding treatment and Evaluating prognosis.

## Materials and Methods

### Data source

A non-systematic review of the literature was performed by screening PubMed up to 1st of August 2019. Using the search terms including "artificial intelligence", "machine learning", “artificial neuron networks”, “deep learning”, "kidney Disease", "chronic kidney disease" , "acute kidney Injury" and “nephrology”.

### Study selection

Literature was derived from English articles or articles that could be obtained with English abstracts. Studies of human dataset were included. References were also identified from the bibliographies of identified articles and the authors' files.

## The function of AI in kidney disease: Alerting systems, Diagnostic assistance, Guiding treatment and Evaluating prognosis

### Alerting systems

Early prediction of deterioration can play an important role in supporting health care professionals, as an estimated 11 percent of hospital deaths follow a failure to promptly recognize and treat deteriorating patients 5.AI can identify information quickly and effectively, explore intrinsic relationship.

#### Alerting AKI

It is reported that AI technology has an advantage in warning of critical illness, such as acute kidney injury (AKI) 6. AKI is a common critical illness in clinic, especially for elderly and perioperative patients. The incidence of AKI was 7-18% among hospitalized and 50% in ICU patients 7, and it was increasing by 11% per year 8. AKI would prolong hospitalization and increase the cost of treatment 9. Patients with AKI would be more likely to progress to end-stage renal disease (ESRD) than those without AKI 10. There were approximately 2 million patients died of AKI every year. The mortality rate was 10-30% for AKI patients without complication, and 30-80% for those with multiple organ failure 11. At present, the diagnosis of AKI is still based on serum creatinine and urine output, which are not obvious in early AKI. It is difficult for clinicians to recognize AKI in time 12. Early recognition and prevention of potential AKI is importance.

The traditional linear models require the statistical assumption of a liner relationship between the covariates and the risk of morbidity, and are often overfitting and multicollinearity. Machine learning approaches were introduced for better or comparable predictive ability than statistical analysis to predict postoperative outcomes. AI may offer opportunities for identifying patients at risk within a time window that enables early treatment 5. On 9 June 2014, National Health Service (NHS) England published the national AKI algorithm in its patient safety alert, recommending “the wide scale introduction and uptake of an automated computer software algorithm to detect AKI” 13. In 2015, Google developed the Streams program, which could predict AKI and send warnings to doctors to early intervention 13. After that, the application of AI in AKI gradually attracted scientists' attention.

Tomaše et al. build a model which can predict 55.8% of all inpatient episodes of AKI, and 90.2% of all AKI that required subsequent administration of dialysis by AI in 703,782 adult patients 5. Lee et al. 14 retrospectively reviewed 2,010 patients after cardiac surgery and used six machine learning techniques, including decision tree, random forest(RF), extreme gradient boosting, support vector machine(SVM), neural network classifier, and deep learning, to train AKI prediction models. The study demonstrated that the machine learning technique of extreme gradient boosting showed significantly better performance than the traditional logistic regression analysis or previous risk scores in predicting both AKI of all stages and stage 2 or 3 AKI after cardiac surgery which may help to evaluate the risk of AKI at the end of surgery. Yin et al.^15^ retrospectively reviewed 8,800 patients undergoing contrast administration to develop the model for prediction of Contrast-induced nephropathy (CIN), the third cause of all hospital-acquired renal failure 16, by the machine learning method of RF. The model shown good predictive ability of CIN development and might provide preventative measures for CIN. Hamid et al. 17 used boosted ensembles of decision trees to train an AKI prediction tool on retrospective data taken from more than 300,000 inpatients. The prediction tool offered important predictive capability for detecting which patients were likely to suffer AKI. Prediction is improved for the cases with closer kinetics to AKI. These tools allow clinicians to potentially intervene before kidney damage manifests. Tang et al. 18 built risk prediction models for AKI in 157 severely burned patients, and compare the prediction performance of XGBoost machine learning and logistic regression model; machine learning method was found to have a better prediction performance than logistic regression. Besides, a Gradient Boosting Machine algorithm predicted serum creatinine-based Kidney Disease Improving Global Outcomes stage 2 AKI using electronic health record data for longitudinal use in 121,158 hospitalized patients. The algorithm had a sensitivity of 84% and a specificity of 85% for stage 2 AKI which the AUC (95% CI) was 0.90 (0.90-0.90) for predicting stage 2 AKI within 24 hours and 0.87 (0.87-0.87) within 48 hours. The AUC was 0.96 (0.96-0.96) for receipt of renal replacement therapy in the next 48 hours 19. Another study demonstrated that machine learning models (multivariate logistic regression, RF and artificial neural networks (ANN)) can predict AKI onset following ICU admission in 23,950 patients with a competitive AUC (mean AUC 0.783) 20 (Table 1).

#### Alerting CKD

There are also reports of AI applications in alerting the occurrence of CKD. A pilot program using e-technologies to detect CKD was conducted in Australia (Electronic Diagnosis and Management Assistance to Primary Care in Chronic Kidney Disease; EMAP-CKD). The software was built on algorithms trained to identify at-risk patients and to order a relevant screening test for CKD 21.

In addition, AI has also been studied in the early warning of complications of CKD. Galloway et al. reported that using only 2 ECG leads, a deep-learning model detected hyperkalemia in patients with renal disease with an AUC of 0.853 to 0.883. The application of artificial intelligence to the ECG may enable screening for hyperkalemia of CKD patients. However, the study is retrospective and need prospective testing. Additionally,the model is a screening test with low specificity, with upwards of 42% false-positive results, which may cause anxiety and inconvenience for patients.^22^. Pilia et al. also use an artificial neural network to reconstruct the extracellular ionic concentrations for both potassium and calcium with an acceptable precision in CKD patients 23.

Besides, AI can also predict cost and mortality of patients. Lin et al. claimed that applying AI modeling could help to provide reliable information about one-year outcomes following dialysis in the aged and super-aged populations. They concluded that those with cancer, alcohol-related disease, stroke, chronic obstructive pulmonary disease (COPD), previous hip fracture, osteoporosis, dementia, and previous respiratory failure had higher medical costs and a high mortality rate 24.

Moreover, Eiichiro et al. identified factors of progressive CKD from healthy population at a health check point by using Bayesian network and artificial intelligence. They included hypertension, the time-series changes in the prognostic category of CKD, proteinuria and eGFR et al 25. Besides, Almansour et al. compared the ANN and SVM techniques in a dataset of 400 patients to predict CKD in early stage. The empirical results from the experiments indicated that ANN performed better than SVM, with accuracies of 99.75% and 97.75%, respectively 26. Chen Z et al. also used multivariate models, i.e., K-nearest neighbor (KNN), SVM, and soft independent modeling of class analogy (SIMCA), to evaluate risk of 386 patients to predict CKD. The overall accuracies were over 93% 27. Bermudez-Lopez M et al. also used RF analysis to point out that new parameters such as Proprotein convertase subtilisin-kexin type (PCSK9) have a higher discrimination ability to classify patients into the non-diabetic CKD group 28.

The high morbidity rate associated with kidney stone disease is one of the main concerns in healthcare systems. Kazemi Y et al. developed a model for the early detection of the type of kidney stone and the most influential parameters in 936 patients with nephrolithiasis. The final ensemble-based model (with an accuracy of 97.1%) could be safely applied to predict the chances of developing nephrolithiasis 29.

Whereas, the current studies are mainly retrospective analyses, and the applicability needs further verification. Moreover, it is reported that an ANN model using 3 variables did not perform better than a new regression model in improving GFR estimation 30. Furthermore, AI technologies face ethical and legal challenges yet to clarify. In 2016, DeepMind Technologies Limited, a wholly-owned subsidiary of the Google conglomerate, Alphabet Inc., announced its first major health project: a collaboration with the Royal Free London NHS Foundation Trust, to assist in the management of AKI. Initially received with great enthusiasm, the collaboration has suffered from a lack of clarity and openness, with issues of privacy and power emerging as potent challenges as the project has unfolded 13. In the end, the project was halted due to a lack of privacy and consent to transferring population-derived dataset to large private prospectors 31.

### Computer Aided Diagnosis--diagnostic assistance

Computer Aided Diagnosis (CAD) is a technology combined medical image and computer image processing to quantify and judge the characteristics of the focus, could assist clinicians to identify and analyze lesions timely and accurately 32. The function of CAD has been verified in many aspects, especially in tumors, such as skin cancer, breast cancer, lung cancer, and so on^33^. The studies related to kidney disease are scanty, mainly about imaging diagnosis and pathological diagnosis.

#### Imaging diagnosis

Autosomal Dominant Polycystic Kidney Disease (ADPKD) is the most common hereditary disease in kidney. It is characterized by progressive enlargement of the kidneys caused by progressive development of renal cysts, often accompanied by declining renal function 34. Total kidney volume (TKV) associated with renal function, is a crucial biomarker for studying progression of ADPKD. Traditional methods for TKV computation are based on stereology and manual segmentation for Computed Tomography (CT) and Magnetic Resonance Imaging. The accuracy of this method depends on user-specified parameters. It is significantly important to develop rapid and reliable methods for TKV quantification, CAD is a good choice. In 2017, Kanishka et al. 35 used an automated segmentation method based on deep learning for TKV computed on CT dataset of 244 ADPKD patients. The new method facilitates fast and reproducible diagnosis, and TKV measurements transmission agrees with manual segmentations from clinical experts. Similarly, Timothy et al. [36]used an automated method to segment the kidney and measure TKV from 2400 cases of MR images. The method simulated a multi-observer approach to create an accurate and robust method for segmentation and computation of TKV. However, CAD technology only could obtain a primary diagnosis. If some characteristics are not included in training database, they need to be judged by the clinician, after that they will be included in the training model to continue learning to improve the diagnostic ability. Most recently, van Gastel MDA et al. successfully developed a fully automated segmentation method for TKV measurement that uses a deep learning network in 540 abdominal magnetic resonance images (T2-weighted HASTE coronal sequences) from patients with ADPKD. TKV measured by the automated approach correlated highly with manually traced TKV (intraclass correlation coefficients, 0.998), with low bias and high precision (<0.1%±2.7% for TKV) 37.

It is often difficult for physicians to achieve preoperative differential diagnosis between renal cell carcinoma and some benign renal tumors by virtue of existing imaging techniques (including CT and MRI), such as adiposity angiomyolipoma 38.In most cases, such benign tumors only require conservative treatment or follow-up, so it is particularly important to achieve accurate preoperative diagnosis of renal cell carcinoma and renal benign tumors^39^. Automatic deep feature classification (DFC) method can distinguished both benign angiomyolipoma without visible fat (AMLwvf)^40^, ^41^ and oncocytoma^42^ from malignant renal cell carcinoma (ccRCC) from abdominal computer tomography (CT) images.

Besides, Image genomics can complete genomic analysis in characterizing disease biology by extracting a large number of tumor image features through AI, and associating the image features with the underlying mutation status of tumors, molecular markers, underlying activated biologic pathways, or clinical outcomes by developing “association maps” between them 43, 44. Jamshidi et al. constructed an alternative model for multi-gene expression molecular detection of renal clear cell carcinoma based on image genomics by using CT imaging features, thus achieving the independent prediction of disease-related survival of patients without any invasion method 45. It is found out that the associations between tumor angiogenesis and radiomic imaging features from PET/MRI, which can predict the prognostic and guide the treatment of antiangiogenic agents of clear cell renal cell carcinoma (ccRCC) 46, 47. However, most of the current clinical studies are single-center studies with small sample size, and lacking cross-test and verification.

What is more, Kuo et al. identified the CKD status defined by an eGFR of <60 ml/min/1.73 m2 based on 4,505 kidney ultrasound images by deep neural network (The AUC of the model is 0.904). A Pearson correlation coefficient of 0.741 and accuracy of 85.6% indicated the strong relationship between AI and creatinine-based GFR estimations 48.

Congenital abnormalities of the kidney and urinary tract (CAKUT) in children are a challenging task. A pre-trained deep learning model (imagenet-caffe-alex) is adopted for transfer learning-based feature extraction from 3-channel feature maps computed from ultrasound images. SVM classifiers are then built upon different sets of features, including the transfer learning features, conventional imaging features, and their combination. The AUC for classifiers built on the combination features were 0.92, 0.88, and 0.92 for discriminating the left, right, and bilateral abnormal kidney scans from controls with classification rates of 84%, 81%, and 87%; specificity of 84%, 74%, and 88%; and sensitivity of 85%, 88%, and 86%, respectively. It is suggested that the combination of transfer learning features and conventional imaging features yielded the best classification performance for distinguishing CAKUT patients from normal controls based on their ultrasound kidney images 49, 50.

#### Pathological diagnosis

Renal interstitial fibrosis is an indicator of the presence and extent of chronic kidney disease. The standard quantitative method of visual evaluation is very important for the diagnosis of renal diseases. The Banff schema is one of the standards used for the renal allograft rejection grades classification. The visual scoring is subject to pathologists' intra- and inter-observer variability, may not be repeatable or reproducible. CAD can reduce the workload of pathologists, also has an advantage of precision and meticulousness. Wei et al.^51^ developed an automated quantification system for measuring the interstitial fibrosis in 40-image and use 70 kidney patients to prove the error rate. It had shown an average error of 9%. The system demonstrated to be an effective quantification system as a diagnostic aide. Kannan et al. developed a deep learning framework to accurately identify and segment glomeruli from digitized images of human kidney biopsies. The segmentation model that was based on the convolutional neural network (CNN) multilabel classifier accurately marked the globally sclerosis glomeruli on the test data, which indicated the power of deep learning for assessing complex histologic structures from digitized human kidney biopsies 52. Vijaya B Kolachalama et al. also leveraged a deep learning architecture to better associate patient-specific histologic images with clinical phenotypes (training classes) including CKD stage, serum creatinine, and nephrotic-range proteinuria at the time of biopsy, and 1-, 3-, and 5-year renal survival in 171 patients. AUC values for the CNN models in predicting creatinine, proteinuria and 1-, 3-, and 5-year renal survival were 0.912, 0.867, 0.878, 0.875, and 0.904 53. Li C et al. trained a RF algorithm in 222 patients to build a pathological prediction model of primary nephrotic syndrome (accuracy 62.2%) 54. However, the specimen number was small, and the general applicability was uncertain, more researches were needed to improve in the future.

#### Make appropriate ICD Codes

International Classification of Disease (ICD) codes is important for population health and cohort discovery when clinical information is limited. Sina Rashidian et al. used deep learning methods which based on demographics, lab results, and medications, as well as codes from previous encounters to model coder decision making. Three test cases were investigated including acute renal failure (ARF) and CKD, the AUC of predicting ARF and CKD were 0.9194 and 0.9424 separately^55^.

### Guiding treatment

Guidelines are the basis for decision-making, which are formulated through large-scale investigations. These guidelines are thus population-based and adjustments are needed using the guidelines on individual cases. Personalized and accurate treatment protocols are required. AI can analyze the association of treatment protocols and efficacy from a large number of patients, develop models based on efficacy and risk factors, guide the choice of treatment protocols, and improve clinical efficacy. Related studies on kidney disease are scanty, mainly in hemodialysis patients.

#### Anemia treatment

Anemia is one of the main common comorbidities in patients undergoing hemodialysis. The incidence and severity of anemia are gradually increasing as renal function declining 56. In 2016, China Dialysis Outcomes and Practice Patterns Study (China's DOPPS) showed the prevalence of anemia in CKD being 21% 57. Anemia can increase the proportion of left ventricular hypertrophy, cause heart failure and myocardial infarction, reduce quality of life and increase the risk of death. Treatment costs and mortality were also significantly increased by anemia 58. The major cause of anemia in CKD is deficiency in erythropoietin (EPO) production 59. Erythropoietin stimulating agents (ESAs), which would supplement EPO and increase hemoglobin (Hb) level, are widely applied by clinicians. However, the toxicities of ESAs have been verified. ESAs would increase the incidence of cardiovascular events, tumor progression, and mortality 60, 61. It was reported that the toxicities are associated with dose 62. It is important to find an adequate treatment for every patient in each particular situation.

Substantial progress has been made in the application of computer-driven methods to provide erythropoietic dosing information for patients with anemia resulting from chronic kidney disease. Initial solutions were simply computerized versions of traditional paper-based anemia management protocols. True personalization was achieved through the use of advanced modeling techniques such as artificial neural networks, physiologic models, and feedback control systems 63. Early in 2003, experts in Spain have carried out an individualized prediction of the EPO dosage to be administered to CKD patients and successfully aided in the individualization of dosage and provided state-of-the-art regression models to clinicians 64 in a single center. In 2008, Gaweda et al. also reported that model predictive control (MPC) by using an artificial neural network model of EPO may result in improved anemia management 65. Both studies had relatively small sample sizes. In 2014, Carlo used Machine Learning (Multilayer Perceptron, MLP) and linear model, which were built for prediction of ESAs therapy response, to recommend suitable ESAs doses. The accuracy of MLP prediction model was more than 90%. The MLP model outperforms previous approaches of the Hb prediction 66. To evaluate the impact of the model, the team conducted a 24-month retrospective study using additional 752 hemodialysis patients in 2016. It was reported that median ESAs consumption decreased and on-target Hb values increased based on the model suggestions. Moreover, Hb fluctuation had a significant decrease. The model could help to improve anemia outcomes of patients with hemodialysis, minimizing ESAs dose with the potential to reduce treatment cost 67. In 2018, María et al. 68 also assessed the usefulness of this model. The model could help to increase the percentage of Hb in range and reduce the intake of ESAs with less Hb fluctuations. Meanwhile, the transfusion, hospitalization and cardiovascular events were all decreased. In conclusion, the model was an effective tool to help clinicians to minimize the risks of treatment with ESAs and reduce costs. However, the sample size was not very large and the follow-up time was insufficient to assess the impact of the model on cardiovascular morbidity and mortality. At that time, the model has only used for hemodialysis patients, more trials should be carried out to verify the usefulness of anemia treatment in pre-dialysis and peritoneal dialysis patients. Moreover, these approaches are data-intensive and typically perform well in ranges in which adequate data exist to model the ESA response, but are unable to extrapolate beyond these ranges 63.

#### Blood pressure and fluid volume management

Blood pressure (BP) and fluid volume are crucial points for patients undergoing hemodialysis. The prevalence of hypertension is 40-90% of patients with ESRD according to the BP definition used, the population selected, and the timing of measurement 69, 70. Clinicians often reduce extracellular fluid volume overload to control BP, which would increase the incidence of intradialytic hypotension. Both intradialytic hypotension and chronic hypertension are associated with poor prognosis. The clinical system EuCliD® is an international electronic health record repository allowing point-of-care data collection of routine clinical practice information 71. By exploiting such wealth of information, Carlo et al. 71 developed a multiple-endpoint model predicting session-specific Kt/V, fluid volume removal, heart rate, and BP based on 766,000 records in 2019. The accuracy and precision of the model is encouraging. The model may help to make the optimized decision in multidimensional other than currently limited single-endpoint treatment strategies.

#### Wearable dialysis devices

**Figure 1:**
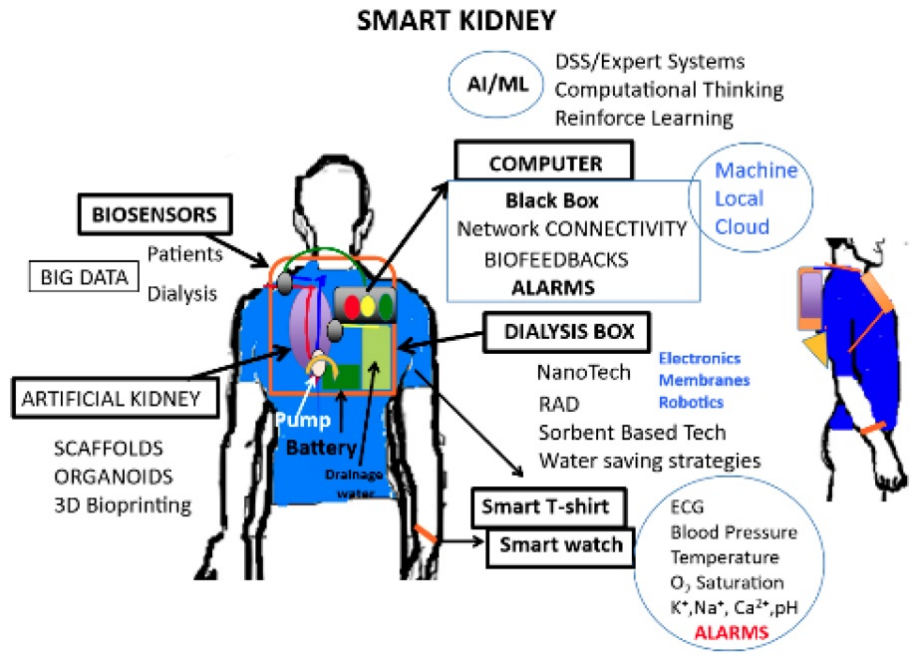
Artificial intelligence involves the science and engineering for developing smart wearable artificial kidneys 72

Dialysis is the main treatment for ESRD. Dialysis affects the patient's life in a wide range, and some patients cannot tolerate hemodynamic instability of intermittent dialysis, there are great expectations for the development of wearable artificial kidneys. Wearable dialysis devices can make the real-time analysis of equipment alarms, dialysis parameters, and patient-related data with a real-time feedback response. Martin et al. 72 combined AI and regenerative medicine technology to develop wearable dialysis devices. These devices can conduct continuous dialysis, remove toxins effectively and have little effect on hemodynamic (Figure 1). The devices have been tested in 15 ESRD patients. The results showed that dialysis was effective without any adverse reactions. The devices were authorized as a breakthrough device by the America Food and Drug Administration. However, the sample size is small; more researches are needed to include more patients to improve the model in the future. Another interesting approach is the wearable artificial kidney, a 5-kg wearable, miniaturized device with a sorbent-based hemodialysis system that is worn on the waist like a toolkit belt and is currently under development at the University of Washington, USA. An exploratory clinical trial with 10 patients received therapy with a wearable artificial kidney for 24 h. However, the trial was stopped after the inclusion of the 7th subject because of device-related technical problems, including an excessive presence of carbon dioxide bubbles in the dialysate circuit, which exceeded its degassing capacity; tubing kinks; and variable pump function that resulted in fluctuating blood and dialysate flow rates 73. Another remarkable innovation is the implantable Renal Assist Device (iRAD) that uses micromachining techniques to fabricate a biohybrid system able to mimic renal morphology and function. This artificial kidney is a bionic device that incorporates a silicon nanopore membrane and a bioreactor of live kidney cells that will concentrate the ultrafiltrate into urine. The bundle is enclosed in a body-friendly box and connected to a patient's circulatory system and bladder. Despite having been used with success in animal models 74, it is still under development to scale it up to the clinical environment 75. Recently, experts found MXene Sorbents for Removal of Urea from Dialysate, which open a new opportunity in designing a miniaturized dialysate regeneration system for a wearable artificial kidney 76.

#### Assistance of needle insertion

**Figure 2:**
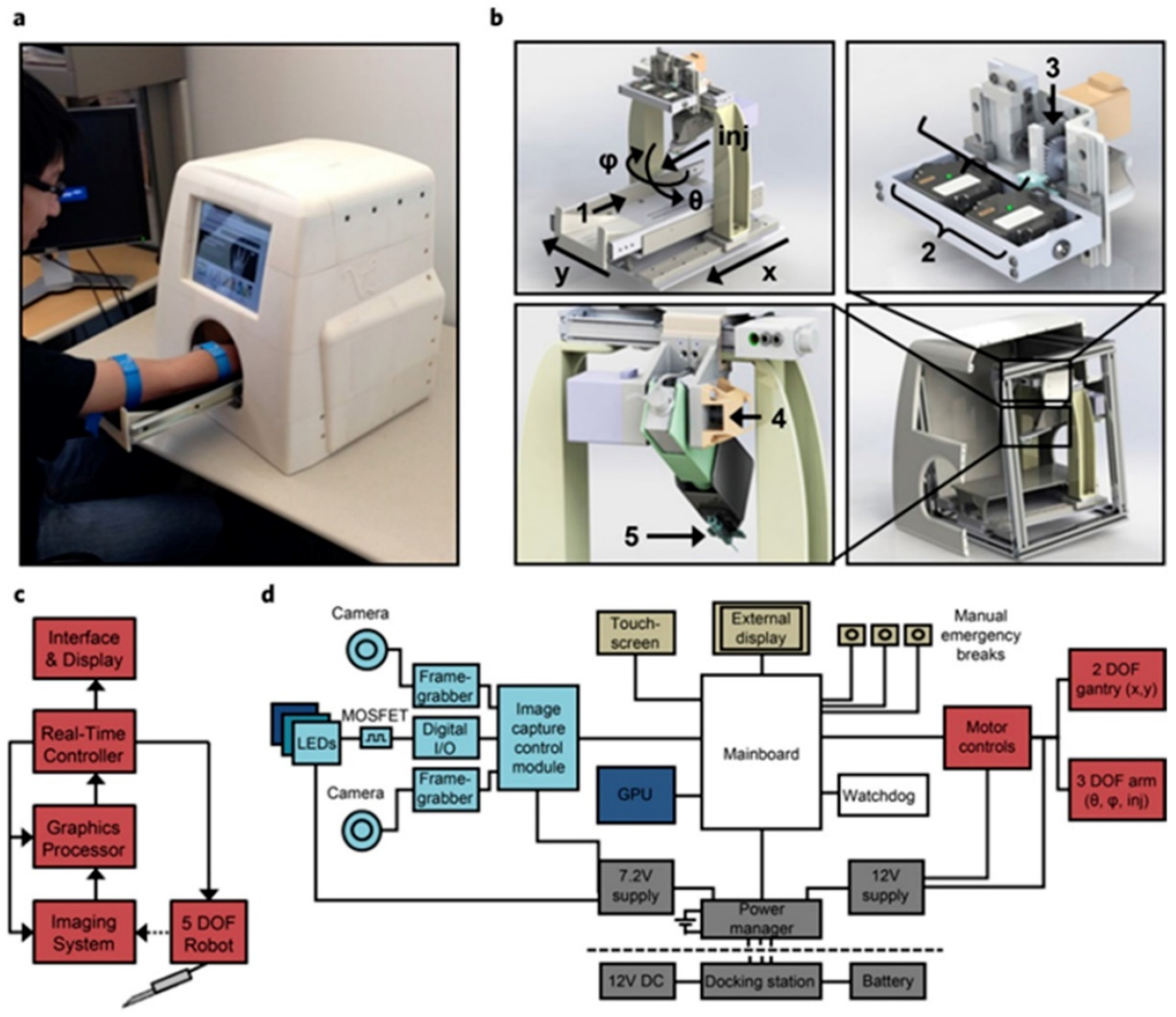
System design and architecture for automated cannula insertion. (a) Functional prototype. (b) Major functional components. (c) Device data flow.(d) Hardware architecture grouped by function 77.

Robotics are also steadily being introduced in health care, especially in surgery, with systems such as the da Vinci® Surgical System that involves a magnified 3D high-definition computer vision system (part of it aided by ML) and tiny wristed instruments that bend and rotate more complexly than the human hand. An autonomous image-guided robotic needle insertion for blood draws and intravenous insertions has also been designed that combines robotics, AI, computer vision, and image technology (Figure 2) 77. It must be made clear that a dialysis machine is not an AI-guided robot, since it is not able to respond to its environments in ways that humans have not explicitly taught it to. However, it is easy to imagine future dialysis robots capable of carrying out complex series of actions automatically or in a semi-autonomous fashion. Besides, it is reported that accurate needle insertion (< 3 mm error) can be achieved in common target sites including the kidneys when using a CT-guided robotic system 78. The proposed optical tracker based robot registration and serving method is capable of accurate three dimension needle operation for ultrasound-guided percutaneous renal access (PRA) procedure with improved precision and shortened time 79.

### Evaluating prognosis

AI can identify factors affecting prognosis by analyzing database, and develop models evaluating the relationship between factors and prognosis. In this section, we will summarize AI applications in evaluating prognosis involving several common kidney diseases.

#### Chronic Kidney Disease Mineral and Bone Metabolism Disorders (CKD-MBD)

CKD-MBD is another common comorbidity in ESRD patients, which increases mortality. The serum concentrations of phosphate (P), calcium (Ca) or parathyroid hormone (PTH) are associated with negative outcomes. The three parameters, P, Ca, PTH, interplay of each other, but the relationship is non-linear. Classical statistical methods have no advantages on analyzing associations among variables non-linear but affected by non-trivial feedback loops. The machine learning seems to be a predictive analytic approach. To quantify the degree of association between the three parameters, Mariano et al developed a data analysis system by RF from 1,758 HD patients. Comparing with Classical statistical methods, the power of prediction of the new model was markedly increased 80. In 2018, Kleiman et al.^81^ used RF algorithms to build model predicting risks for development of calciphylaxis in CKD patients.With an AUC value of 0.872, the model could successfully predict calciphylaxis, provide an opportunity for clinical translation of the predictive models.

#### IgA nephropathy (IgAN)

IgAN is the most common primary glomerular kidney disease. About 20-40% of IgAN patients will reach ESRD within 10-20 years of diagnosis. Early prediction of ESRD is valuable. Liu et al. 82 retrospectively reviewed 262 biopsy-proven IgAN patients, and employed artificial intelligence to predict the ESRD status in IgAN patients. The predictive model showed that Oxford-MEST scores, C3 staining and eGFR were important role for ESRD prediction in Chinese IgAN patients. Most recently, Chen et al. 83 developed a prediction model using routinely available characteristics (demographic, clinical, and pathologic variables) and based on the combination of a machine learning algorithm and survival analysis which can stratify risk for kidney disease progression in the setting of IgAN.

#### Diabetic Kidney Disease (DKD)

DKD is a common complication and a mainly cause of diabetes deaths. It is critical to identify the risk factors to stratify DKD risk to improve patient management. As early in 2013, Leung RK et al. used a multi-staged strategy based on machine learning and mathematical modeling to predict genotype-phenotype risk patterns in 119 DKD patients and 554 without DKD type 2 diabetic patients. They found out that age, age of diagnosis and lipid parameters were major clinical predictors while genetic polymorphisms related to inflammation and lipid metabolism were the most selected genetic predictors 84.

In 2018, Arianna et al. 85 used machine learning to set a predictive of type 2 diabetes mellitus complications from 1000 patients with type 2 diabetes. The Random Forest model had the highest predictive performance. The model showed that variables, such as gender, age, time from diagnosis, body mass index, glycated hemoglobin, hypertension, and smoking habit, contributed more than others. The accuracy of the model was 0.838, more studies should be conduct to improve the accuracy and sensitivity. Another research also claimed that machine learning could compare frequent medical problems of African American and Caucasian Diabetic Kidney disease in 4,623 diabetic patients. This study found that African Americans have much higher rates of CKD-related medical problems than Caucasians for all five CKD stages, and prominent markers leading to ESRD were high glucose, high systolic BP, obesity, alcohol/drug use, and low hematocrit. In 2019, Ravizza et al. 86 carried out a direct comparison of algorithms derived from real-world data (RWD) and clinical data for quantifying the risk of CKD as a long-term complication of diabetes. After teaching the Roche/IBM model using seven prioritized features, including age, body mass index, and glomerular filtration rate, creatinine, albumin, glucose, and hemoglobin, the AUC of the prediction algorithm was 0.7937. It is concluded that the teaching of predictive analytics algorithms using real-world data could achieve equivalent or even enhanced accuracy compared with those using clinical trial data. Most recently, Song X et al. presented an ensemble feature selection approach to identify a robust set of discriminant factors to predict DKD in 15,645 adult patients with type 2 diabetes 87. The ensemble model identified a set of 440 features, including 191 labs, 51 visit details (mainly vital signs), 39 medications, 34 orders, 30 diagnoses, and 95 other clinical features with an AUC of 0.82 on internal validation and 0.71 on external temporal validation. Besides, Makino et al. also constructed a new predictive model for DKD using AI based on the EMR of 64,059 diabetes patients. AI could predict DKD aggravation with 71% accuracy (the resultant average of the AUC was 0.743) which may contribute to more effective and accurate intervention to reduce hemodialysis 88.

#### Kidney transplantation

Kidney transplantation is another main treatment for ESRD. But only a few patients can receive kidney transplantation because the shortage of kidney and high technical requirements. Predicting the outcome of kidney transplantation is important to optimize transplantation parameters and modify factors related to recipient, donor, and transplant procedure. Lofaro et al. 89 retrospectively analyzed 80 renal transplant patients with 5-year follow-up, using classification trees to develop two predictive models and identifying six variables that contributed high on patient outcomes. The AUC values of these two models are 0.847 and 0.824 respectively. In another study, 4754 systemic lupus erythematosus patients who underwent kidney transplantation were enrolled. Predictive models were developed by 3 machine learning algorithm. The AUC of ANN (0.73) based on six variables was slightly better than the logistic regression based on six variables selected by Weka (0.73) and classification trees (0.70). The study showed that the ANN model had better predictive performance than other models 90. Abdeltawab H et al. also developed a deep-learning based CAD system based on the fusion of both imaging markers and clinical biomarkers for the early detection of acute renal transplant rejection. The overall accuracy of the proposed system is 92.9% with 93.3% sensitivity and 92.3% specificity in distinguishing non-rejected kidney transplants from rejected ones 91.

#### CKD after AKI

Several studies between 2008 and 2017 have demonstrated that although acute kidney injury is usually reversible, some patients may experience incomplete recovery of kidney function, while others subsequently develop accelerated loss of kidney function, resulting in an increased risk of chronic kidney disease 92-94. A prediction of advanced CKD following hospitalization with AKI will result in got opportunities to intervene and potentially improve long-term outcomes and avoid unnecessary use of clinical resources 95, 96. A multivariable model was developed with 9973 participants and was externally validated with 2761 participants to develop a practical risk stratification approach that could be used to identify patients at high risk of chronic kidney disease after they are discharged. This model using routine laboratory data was able to predict advanced chronic kidney disease following hospitalization with acute kidney injury. But requires evaluation of its utility in a clinical setting 97.

#### CKD severity

Norouzi et al. predicted renal failure progression in 465 CKD patients by using Integrated Intelligent Fuzzy Expert System and found out that the model could accurately (>95%) predict the GFR for sequential 6-, 12-, and 18-month intervals 98. Xiao et al. compared nine predictive models, including logistic regression, Elastic Net, lasso regression, ridge regression, SVM, RF, XGBoost, neural network and k-nearest neighbor in prediction of CKD progression in 551 patients with proteinuria (The AUC values of the 9 models were 0.873, 0.871, 0.872, 0.865, 0.857, 0.854, 0.868, 0.854, 0.802, respectively). The study showed that the model with the highest sensitivity was Elastic Net (0.85), while XGBoost showed the highest specificity (0.83). ALB, Scr, TG, LDL and eGFR levels, showed predictive ability for CKD severity 99. Moreover, Zacharias HU et al. identified CKD patients at risk of progressing to ESRD in 4,640 patients by state-of-the-art machine learning methods. The results demonstrated that proton nuclear magnetic resonance features, such as creatinine, high-density lipoprotein, valine, acetyl groups of glycoproteins, and Ca^2+^-EDTA carried the highest weights are predicting factors 100.

#### Death Risk, cardiovascular risk after dialysis

The study was carried out on a contemporary cohort of 27,615 US veterans with incident ESRD by implementing a random forest method on 49 variables obtained before dialysis transition to predict outcomes of 30-, 90-, 180-, and 365-day all-cause mortality after dialysis initiation. The results showed that the model provided C-statistics (95% CI) of 0.7185 (0.6994-0.7377), 0.7446 (0.7346-0.7546), 0.7504 (0.7425-0.7583), and 0.7488 (0.7421-0.7554) for predicting risk of death within the 4 different time windows 101. Mezzatesta S et al. used non-linear SVC with RBF kernel algorithm, optimized with GridSearch, allowed to obtain an accuracy of 95.25% in the Italian dataset and of 92.15% in the American dataset, in the prediction of Ischaemic Heart Disease in patients on dialysis 102.

## Summary

**Figure 3:**
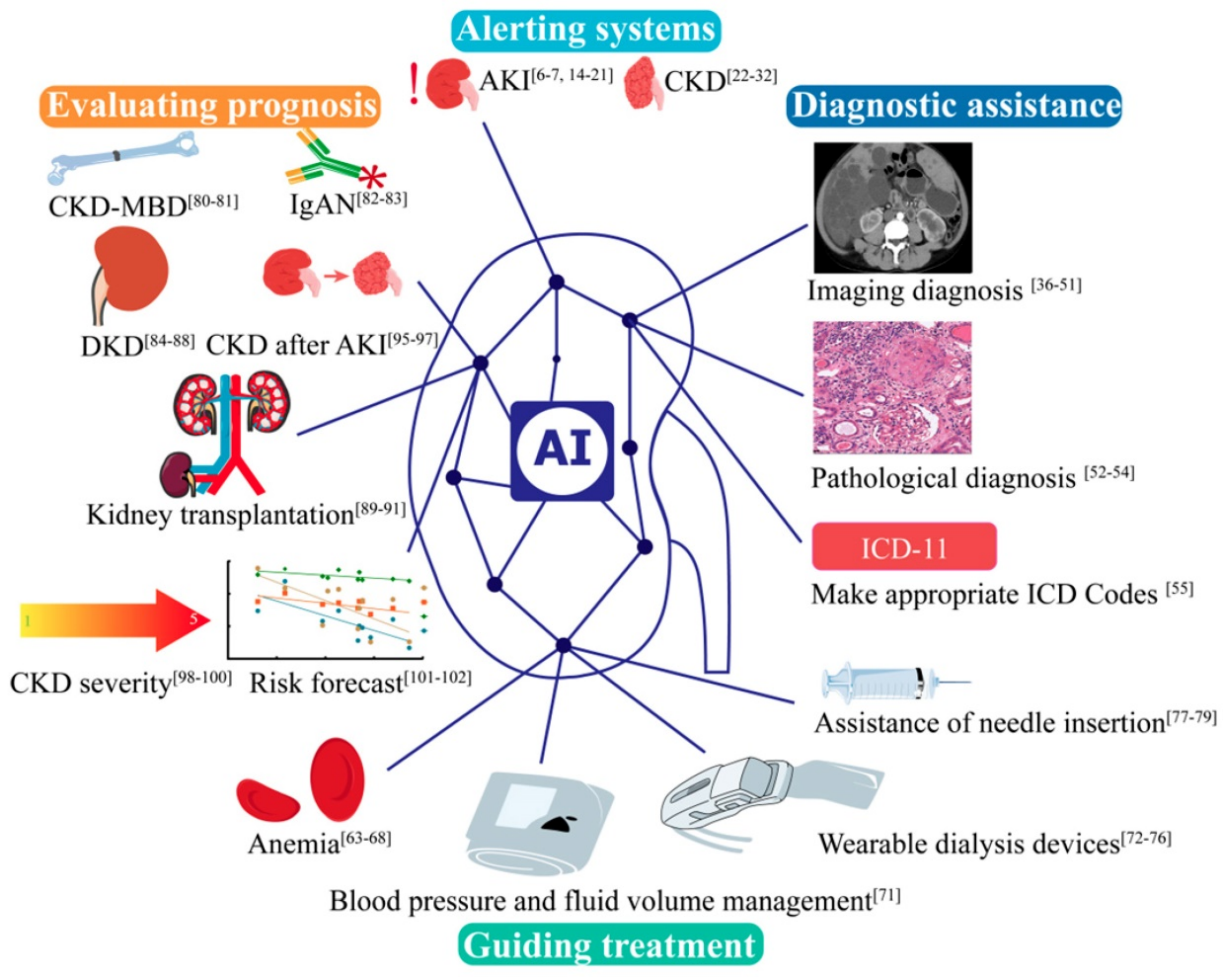
AI applications in kidney disease in alerting systems, diagnostic assistance, guiding treatment and evaluating prognosis. AKI, acute kidney disease. CKD, chronic kidney disease. CKD-MBD, Chronic Kidney Disease - Mineral and Bone Disorder. IgAN, IgA nephropathy.

It is noticeable that AI does not only play great role in Nephrology (Figure 3), but also in many other disciplines. Take Urology as an example, AI is applied for the prediction of genitourinary cancers 103. We are witnessing the development of medical practice from empirical medicine to evidence-based medicine to intelligent diagnosis and to AI-directed medicine. While AI medicine is still in its infant stage, it is no question that by taking advantage of the diversity and complexity of the real-world data, AI will produce prediction algorithms suitable for routine clinical application in the near future. These findings may fuel a fundamental discussion of future medical evidence beyond the exploration of original targets for analyzing and interpreting data that are potentially costly, long-term clinical trials with a limited number of patients that may one day enhance, or even partially replace, real-world data-driven risk assessments 86. In the future, AI is increasingly important in clinic, which can make clinician work efficiency and alleviate the pressure. Nonetheless, AI is facing multiple challenges, including the data quality uneven, lacking of agreed standards of different center, lacking of scientific verification, and the security, privacy of data. At the same time, the current studies are major retrospective studies, and absent large multi-center studies. More research is needed to apply AI to kidney diseases.

## Figures and Tables

**Table 1:** Summary the role of AI in predicting AKI

Study	cohort size	Research Type	AI Algorithm	specificity	sensitivity	AUC	Factors used in the development of individual AI models	Limitations
Tomašev N,et al.^5^	703,782	longitudinal dataset	recurrent neural network	83.3% (dialysis within 30 days)	84.3% (dialysis within 30 days)	83.5%(dialysis within 30 days)	Age, ethnicity, gender, diabetes	Retrospective study. The model is not representative of the global population
Sanchez-Pinto LN,et al.^6^	6,564	analytical study	regression-based methods (stepwise backward selection using p-value and AIC, Least Absolute Shrinkage and Selection Operator, and Elastic Net) and tree-based methods (Variable Selection Using Random Forest, Regularized Random Forests, Boruta, and Gradient Boosted Feature Selection)	NA	NA	AUC of 0.82 for the logistic regression, 0.83 for the random forest, and 0.80 for the gradient boosted machine.	Age, years, Weight, Urine output (UOP), Bilirubin, mg/Dl,Blood urea nitrogen (BUN), Hemoglobin, Platelets, Potassium ,White blood cell count (WBC), Lowest systolic blood pressure(SBP),Systolic blood pressure standard deviation, Lowest SaO2/FiO2 (SF) ratio ,Vasoactive-inotropic score (VIS), Disseminated intravascular coagulopathy (DIC) score, et al.	Retrospective study. The study used the default settings of the algorithms that they tested and made no attempts to optimize the algorithms using different settings.
Lee HC, et al.^14^	2,010	Retrospective study	decision tree, RF, extreme gradient boosting, SVM, neural network classifier	NA	NA	0.55-0.78	Age, Female, Body-mass index, Surgery type, Coronary artery bypass, Valvular heart surgery, Thoracic aortic surgery, Emergency, et al.	Retrospective study. The analysis used only single-center data and included a relatively small number of cases and covariates, the external validity of results may be limited. Important predictors may be different according to different institutions. It is not certain that their results could translate into improved clinical outcomes for the patients.
Yin WJ, et al.^15^	8,800	a retrospective single-center case con- trol study	the machine learning method of random forest	0.788	0.827	0.907	baseline eGFR, red cell distribution width (RDW), triglycerides, the most recent serum creatinine before the procedure, high-density lipoprotein cholesterol (HDL), total cholesterol, low-density lipoprotein cholesterol (LDL), blood urea (BU), platelet larger cell ratio (P-LCR), serum sodium (Na+), plateletocrit (PCT), international normalized ratio (INR), and blood glucose (BG).	This study is limited by its retrospective design; the prediction model is derived and validated by a single center; any variable that was missing for more than 30% of the population was not assessed in the present study; they ignored unstruc- tured clinical notes.
Mohamadlou H, et al.^17^	30,0000	Retrospective study	gradient boosted trees	Prediction at Onset: 0.82. Prediction 12 hours before onset: 0.73. Prediction 24 hour: 0.64 (patient data from Stanford Medical Center)	Prediction at Onset: 0.77. Prediction 12 hours before onset: 0.75. Prediction 24 hour: 0.79(patient data from Stanford Medical Center)	Prediction at Onset: 0.872 Prediction 12 hours before onset: 0.800 Prediction 24 hour: 0.795(patient data from Stanford Medical Center)	Age, gender, heart rate, respiratory rate, temperature, SCr, and Glasgow Coma Scale (GCS), et al.	Retrospective study. They cannot draw any conclusions about the impact the algorithm's predictions will have on patient outcomes in a clinical setting.
Tang CQ, et al.^18^	157	Retrospective study	Logistic regression and XGBoost machine learning algorithm	0.844 (Logistic regression) 0.897(XGBoost machine learning algorithm)	0.777 (Logistic regression) 0.820(XGBoost machine learning algorithm)	0.875(Logistic regression) 0.920(XGBoost machine learning algorithm)	sex, age, admission time, features of basic injuries, initial score on admission, treatment condition, and mortality on post injury days 30, 60, and 90, et al.	Retrospective study. The prediction model in this article needs to be further validated in prospective studies; Do not record the use of nephrotoxic drugs, and do not explore its role in Effects on AKI in the model; the results of this study may be applicable only to patients with severe burns and inhaled injuries. Retrospective study, limited no. of pts
Koyner JL, et al.^19^	121,158	Observational cohort study.	Gradient Boosting Machine algorithm	0.61 (Probability Cutoff≥ 0.004 in Stage 2 Acute Kidney Injury)	0.96 (Probability Cutoff≥ 0.004 in Stage 2 Acute Kidney Injury)	0.87 (0.87-0.87) At Least Stage2 AKI (48 hr)	Demographics, location data, vital signs, laboratory values, interventions, medications, nurse documentation, and diagnostic orders	The study did not use the urine output definitions of AKI due to the inability to accurately measure urine output on an hourly basis in the majority of the cohort, they are limited in that they do not have SCr values prior to the index hospitalization and as such cannot truly determine baseline SCr or know if a patient has CKD. They do not have information on unique procedures that occur during the index hospitalization (e.g., specific surgical procedures) nor do they have baseline comorbidity information.
Zimmerman LP, et al.^20^	23,950	Retrospective study	multivariate logistic regression, random forest, artificial neural networks	0.730-0.756	0.660-0.698	0.783	Gender, Age, Ethnicity, Creatinine, Heart Rate Maximum (bpm), Heart Rate Mean (bpm), et al.	Retrospective study. Data is not missing-at-random. Do not include comorbid diagnoses. Prospective trials with independent model training and external validation cohorts are needed.

NA, not available. pts, patients.

**Table 2:** Summary the role of AI in alerting CKD

Study	cohort size	Research Type	AI Algorithm	specificity	sensitivity	AUC	Factors used in the development of individual AI models	Limitations
Galloway CD, et al.^22^	449,380	Retrospective study	A deep convolutional neural network (DNN)	0.632 for Minnesota,0.547 for Florida; 0.550 for Arizona.	0.902% for Minnesota; 0.913 for Florida; 0.889 for Arizona.	0.883 for Minnesota, 0.860 for Florida, and 0.853 for Arizona.	Age, Sex, BMI, eGFR,ECGs, serum potassium, et al.	Retrospective study, a prospectively validated screening test in the home setting is needed to improve care and outcomes in patients with renal and cardiac disease.
Pilia N, et al.^23^	71	Retrospective study	neural network, Bayesian neural network	NA	NA	NA	concentrations of Ca^2+^and K^+^、ECG, et al.	Retrospective study, limited no. of pts.
Lin SY, et al.^24^	48,153	Retrospective study	RF, ANN	NA	0.817 for RF, 0.640 for ANN	0.861 for RF, 0.685 for ANN	Age, Sex, Urbanization level, Occupation, comorbidity, et al.	No external validation; majority of participants were Taiwanese, further validation with different populations is require; lack of detailed information like clinical frailty scales,routine activities,body mass index, et al.
Kanda E, et al.^25^	7,465	observational and worksite-based study	Bayesian network and SVM	NA	NA	NA	age, gender, body mass index (BMI), waist circumference, systolic and diastolic blood pressures, casual blood glucose, hemoglobin A1c (HbA1c) (NGSP), serum low-density lipoprotein (LDL) cholesterol, and creatinine levels, and proteinuria grade, et al.	The results may be biased by unmeasured confounders; the population mainly consisted of healthy workers, and did not include elderly people and the subjects with missing data in this study; The study was carried out in only one region in Japan.
Almansour NA, et al.^26^	400	Retrospective study	ANN /SVM	99.75% /97.75%(accuracy)	NA	NA	Age , Blood Pressure, Blood Glucose , Blood Urea , Serum Creatinine, Sodium , Potassium, Hemoglobin , Packed Cell Volume , White Blood Cell Count , Red Blood Cell Count, et al.	Retrospective study, limited no. of pts
Chen Z, et al.^27^	386	Retrospective study	K-nearest neighbor (KNN), SVM, and soft independent modeling of class analogy (SIMCA)	99.9% (SVM)	97.6% (SVM)	NA	Age, Blood pressure, Specific gravity, Albumin, Sugar, Red blood cell, Pus cella, Pus cell clumps, Bacteria, Blood glucose random , Blood urea, Serum creatinine, Sodium, Potassium, Hemoglobin , Packed cell volume , White blood cell count, Red blood cell count, Hypertension , Diabetes mellitus, Coronary artery disease, Appetite, Pedal edema, Anemia	Retrospective study, limited no. of pts
Bermudez-Lopez M, et al.^28^	395	Cross-sectional study	RF	NA	NA	0.789	VLDL, cholesterol, triglyceride content in IDLs, LDL , HDL, triglycerides, Lp(a), the triglycerides/HDLCholesterol, PCSK9/LDL- Cholesterol ratios, et al.	Cross-sectional study, limited no. of pts
Kazemi Y, et al.^29^	936	Retrospective study	ensemble-based model	97.1%	97.1%	99.6%	sex, acid uric condition, calcium level, hypertension, diabetes, nausea and vomiting, flank pain, and urinary tract infection (UTI), et al.	Retrospective study, limited no. of pts
Liu X,et al.^30^	1,230	Retrospective study	ANN	0.787 (Accuracies)	NA	NA	age, sex, and standardized serum creatinine level, et al.	Retrospective study. Different methods for measuring GFR were a source of systematic bias in comparisons of new models to CKD-EPI, and both the derivation and validation cohorts consisted of a group of patients who were referred to the same institution
